# Hemodynamic response related to the Airway Scope versus the Macintosh laryngoscope: A systematic review and meta-analysis with trial sequential analysis

**DOI:** 10.1097/MD.0000000000033047

**Published:** 2023-02-22

**Authors:** Takumi Nagumo, Hiroshi Hoshijima, Koichi Maruyama, Takahiro Mihara, Tsutomu Mieda, Aiji Sato (Boku), Toshiya Shiga, Hiroshi Nagasaka

**Affiliations:** a Department of Anesthesiology, Saitama Medical University Hospital, Moroyama, Saitama, Japan; b Division of Dento-Oral Anesthesiology, Tohoku University Graduate School of Dentistry, Sendai, Miyagi, Japan; c Department of Anesthesiology, University Hospital Mizonokuchi, Teikyo University School of Medicine, Kawasaki, Kanagawa, Japan; d Department of Health Data Science, Yokohama City University Graduate School of Data Science, Yokohama, Kanakgawa, Japan; e Department of Anesthesiology, Aichi Gakuin University School of Dentistry, Nagoya, Aichi, Japan; f Department of Anesthesiology and Intensive Care Medicine, International University of Health and Welfare, School of Medicine, Ichikawa, Chiba, Japan.

**Keywords:** airway scope, blood pressure, heart rate, hemodynamics, Macintosh laryngoscope, meta-analysis

## Abstract

**Background:**

It is important to reduce the hemodynamic response during tracheal intubation. We performed a systematic review and meta-analysis of the Airway Scope and Macintosh laryngoscope to determine whether they reduce the hemodynamic responses of heart rate (HR) and mean blood pressure (MBP) after tracheal intubation under general anesthesia.

**Methods:**

We performed a comprehensive literature search of electronic databases for clinical trials comparing hemodynamic response to tracheal intubation. The primary aim of our meta-analyst is to determine if the Airway Scope reduces hemodynamic responses (HR and mean MBP) 60 seconds after tracheal intubation compared to the Macintosh laryngoscope. We expressed pooled differences in hemodynamic responses between the 2 devices as weighted mean differences with 95% confidence intervals. We conducted trial sequential analysis. Secondarily, we investigated the ability of the Airway Scope and Macintosh laryngoscope to reduce hemodynamic responses at 120 seconds, 180 seconds, and 300 seconds after tracheal intubation.

**Results:**

We identified clinical trials comparing hemodynamic response via a comprehensive literature search. Of 185 articles found in the search, we selected 8. In comparison to the Macintosh laryngoscope, the Airway Scope significantly reduced HR and MBP at 60 seconds after tracheal intubation (HR; weighted mean difference = −7.29; 95% confidence interval, −10.9 to −3.62; *P* < .0001; *I*^2^ = 57%, MBP; weighted mean difference = −11.5; 95% confidence interval, −20.4 to −2.65; *P* = .01; *I*^2^ = 91%). At the secondary outcome, the Airway Scope significantly reduced the fluctuation of HR after 120 seconds and 180 seconds of tracheal intubation. However, the Airway Scope did not significantly reduce MBP 120 seconds, 180 seconds, and 300 seconds after tracheal intubation. Trial sequential analysis suggested that the total sample size reached the required information size for heart rate.

**Conclusions:**

Our finding suggested that the Airway Scope attenuated hemodynamic responses at 60 seconds after tracheal intubation in comparison with that of the Macintosh laryngoscope. However, the MBP sample size is small and further research is needed.

## 1. Introduction

Tracheal intubation using the Macintosh laryngoscope is often associated with hypertension, tachycardia, and increased plasma catecholamine concentrations,^[1,2]^ which may greatly increase the risk of myocardial infarction and stroke.^[3–5]^ These complications are particularly important in elderly patients and patients with a history of myocardial infarction or stroke. The mechanism behind these excessive cardiovascular responses is considered to be sympathetic activation due to mechanical stimulation of the upper respiratory tract.^[6,7]^ Accordingly, preventing fluctuations in hemodynamic response is important during tracheal intubation. Various preventive measures have been reported, such as increasing the anesthetic induction dose, applying topical local anesthesia spray to the pharynx, and administering an antihypertensive at the same time as anesthetic induction.^[8–11]^ However, these methods are difficult to adjust because the dose of anesthetic may be excessive, the amount of local anesthetic sprayed may be uncertain, and excessive hypotension or tachycardia may be induced.^[8–11]^

The Airway Scope (Nihon Kohden, Tokyo, Japan) is a single-use optical video laryngoscope for tracheal intubation. The Airway Scope has a charge-coupled device camera, and liquid crystal display monitor built into the Airway Scope main unit. Furthermore, a disposable transparent blade (Intlock) is connected to the Airway Scope main unit for use. The Intlock guides the intubation tube into the glottis and ensures sufficient space in the pharynx for adequate observation of the laryngeal structures. The Airway Scope realizes more accurate tracheal intubation by matching the target mark on the liquid crystal display with the glottis during tracheal intubation.^[12,13]^ The special design of this scope optical components and the curvature of its rigid blade allow intubation without needing to align the anatomical 3 axes, and thus may help in reducing the hemodynamic response during tracheal intubation as less force is exerted on the Oro pharyngeal tissue. However, it is unclear whether the Airway Scope actually attenuates hemodynamic response during tracheal intubation compared to the ML. Although some clinical studies have found that the Airway Scope reduces hemodynamic response compared with the ML during tracheal intubation,^[14–16]^ other studies have shown no reduction in hemodynamic response with the Airway Scope.^[17]^

To investigate this issue, we performed a systematic review and meta-analysis of the Airway Scope and the ML to determine whether they reduce the hemodynamic responses of heart rate (HR) and mean blood pressure (MBP) at 60 seconds after tracheal intubation under general anesthesia, and whether the Airway Scope reduces hemodynamic response at 120 seconds, 180 seconds, and 300 seconds after tracheal intubation in comparison with the ML.

## 2. Methods

The manuscript was prepared in accordance with the guidelines recommended by the preferred reporting items for systematic reviews and meta-analyses statement.^[18]^ This meta-analysis registered before the start of the study with the study protocol with the UMIN Clinical Trials Registry (Registration number: UMIN 000031573). Our study is a meta-analysis and does not require ethics committee approval.

### 2.1. Inclusion and exclusion criteria

Our meta-analysis included, as inclusion criteria, adult patients who underwent tracheal intubation with Airway Scope, and the ML under general anesthesia. In addition, a study compared HR and MBP during tracheal intubation was included in our study. Our meta-analysis excluded studies that did not compare HR and MBP during tracheal intubation, studies using double-lumen tubes for tracheal intubation, and studies of tracheal intubation in children.

We have the following PICO (Patient/Problem/Population; Intervention/Exposure; Comparison and Outcomes) of present meta-analysis.

1. Population: Patients undergoing general anesthesia with tracheal intubation.
2. Interventions: Tracheal intubation using the Airway Scope.
3. Comparisons: Tracheal intubation using the ML.
4. Outcomes: Changes in hemodynamic responses (HR and MBP) before and after tracheal intubation.

### 2.2. Search strategy

We searched for eligible trials in the PubMed, EMBASE, and the Cochrane Central Register of Controlled Trials Scopus. We additionally searched references manually. There are no restrictions on the language of the article, and the most recent search was performed in June 2022. The search strategy is provided in Supplemental Digital Content 1, http://links.lww.com/MD/I521.

### 2.3. Selection of included studies

#### 2.3.1. Data extraction.

We selected the trials to include in our meta-analysis by title and summary or abstract. After scanning, we read the full text to assess whether the selected studies met the inclusion criteria in our meta-analysis. These works were performed by 2 different authors. Trials that met the inclusion criteria were independently assessed by each author using standardized data collection forms. Disagreement between the authors was resolved through discussion. In the event of suspected data discrepancies, we contacted the relevant author directly.

### 2.4. Critical appraisal of study quality

#### 2.4.1. Risk of bias assessment.

We used Cochrane risk of bias tool to assess the quality of the study design and the degree of potential bias (Supplemental Digital Content 2, http://links.lww.com/MD/I522).^[19]^ HH and TS independently evaluated the risk of bias, and when they did not agree, they decided by discussion.

We applied the grading of recommendations assessment, development and evaluation approach^[20]^ with grading of recommendations assessment, development and evaluation pro software (version 3.6, https://www.gradepro.org/) to assess the quality of evidence of the main outcomes (Supplemental Digital Content 3, http://links.lww.com/MD/I523).

#### 2.4.2. Data synthesis and analysis.

We performed statistical processing using Review Manager software (version 5.2, Nordic Cochrane Centre, The Cochrane Collaboration, Copenhagen, Denmark). A random-effects model was used to calculate weighted mean difference (WMD) and corresponding 95% confidence interval (CI) for the data. We also tested heterogeneity using the Cochran Q statistic and the *I*^2^ statistic.^[21]^

We performed the sensitivity analysis using a multivariate random-effects model accounting for within-study correlation of the longitudinal data. We used the “metaphor” package in the R statistical computing language for the sensitivity analysis.^[22,23]^

Then, we conducted trial sequential analysis (TSA) to assess sensitivity. This analysis can prevent type I error due to multiple testing of the effect in the meta-analysis.^[24–29]^ First, we calculated the required information size (RIS). We set the risk of type I errors at 5% and the risk of type II errors at 10%. Minimum clinically meaningful mean differences of 10 beats per minute for HR and 5 mm Hg for MBP were used for the TSA. We performed TSA calculation using TSA viewer (version 0.9.5.9 beta; www.ctu.dk/tsa).

To assess the public bias, we tested the symmetry by funnel plot^[30]^ and evaluated the symmetry of funnel plot by Begg test.^[31]^ The existence of public bias is recognized, when the *P* value of Begg test is < 0.1. However, we did not evaluate publication bias when fewer than 9 studies were included in the analysis.

## 3. Results

### 3.1. Characteristics of studies included in the meta-analysis

**Figure 1.**
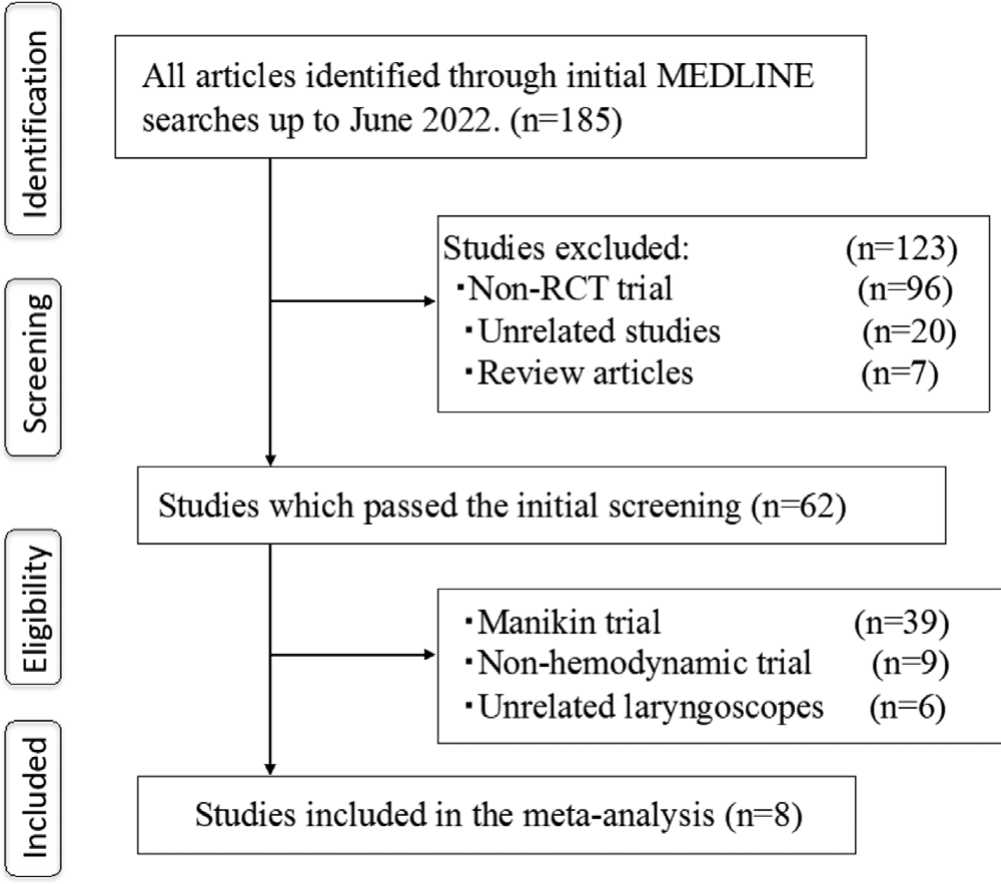
Meta-analysis flow chart. RCT = randomized controlled trial.

We initially identified 185 articles for review from our search of the electronic databases. Of these, we excluded 123 studies that were not randomized controlled trials, were unrelated to the present investigation, or were review articles. We then thoroughly read the remaining 62 articles to determine whether they met the criteria for inclusion. Of these 62 studies, 54 were excluded because they were manikin studies (n = 39), did not investigate hemodynamic responses (n = 9), compared unrelated laryngoscopes (n = 6), or a double-lumen tube was used (n = 2). We ultimately identified 8 trials satisfied our inclusion criteria (Fig. 1).^[14–17,32–35]^ Overall, 302 and 297 tracheal intubations were performed with the Airway Scope and the ML, respectively.

Details of these 8 trials are listed in Table 1. In our meta-analysis, most of trials were research of American Society of Anesthesiologists-Physical Status classification I to II. Only 1 study included studies with American Society of Anesthesiologists-Physical Status classification II to III. In addition, our research included 3 studies of tracheal intubation in the normal airway, 2 studies assuming manual in-line neck stabilization, and 2 studies of predict difficult airway. All tracheal intubations were performed by oral intubation. Most studies used propofol as an anesthesia-inducing drug, but only 1 study used midazolam.

**Table 1:** Characteristics of assessed studies.

	First author	Publication year	AWS/Mac	ASA-PS	Preoperative airway assessment	Intubation pass way	Surgery	Induction of anesthesia	Measuring method of blood pressure
1	Malik M. A	2008	30/30	I–III	MILS	Oral intubation	Elective surgery	Propofol 2–4 mg/kg atracurium 0.5 mg/kg fentanyl 1–1.5 μg/kg	Non invasive
2	Malik M. A	2009	30/30	I–III	MILS	Oral intubation	Elective surgery	Propofol 2–4 mg/kg atracurium 0.5 mg/kg fentanyl 1–1.5 μg/kg	Non invasive
3	Malik M. A	2009	25/25	I–III	Predict difficult airway	Oral intubation	Elective surgery	Propofol 2–4 mg/kg atracurium 0.5 mg/kg fentanyl 1–1.5 μg/kg	Non invasive
4	Nishikawa K	2009	20/20	I–II	Normal	Oral intubation	Mastectomy or minor orthopaedic surgery	Propofol 1.5 mg/kg vecuronium 0.1 mg/kg fentanyl 1 μg/kg	Non invasive
5	Koyama Y	2011	23/23	I–II	Normal	Oral intubation	Elective surgery	Propofol 1.5 mg/kg vecuronium 0.15 mg/kg fentanyl 2 μg/kg	NR
6	Woo C.H	2012	50/50	II–III	Difficult airway	Oral intubation	Escharotomy surgery	Propofol 1.5 mg/kg rocuronium 0.8 mg/kg	Non invasive
7	Kanchi M	2015	15/15	I–II	Normal	Oral intubation	Coronary artery bypass graft	Midazolam 0.1–0.2 mg/kg pancuronium 0.1 μg/kg fentanyl 5–10 μg/kg	Invasive
8	Kim K. N	2017	110/110	I–II	N/A	Oral intubation	Elective surgery	Propofol 1.5 mg/kg rocuronium 0.6 mg/kg, remifentanil 0.1µg/kg/min	NR

ASA-PS = American Society of Anesthesiologists-Physical Status classification, AWS = Airway Scope, Mac = Macintosh laryngoscope, MILS = manual in-line neck stabilization, NR = not recorded.

### 3.2. Results of the meta-analysis

#### 3.2.1. Primary outcome.

**Figure 2.**
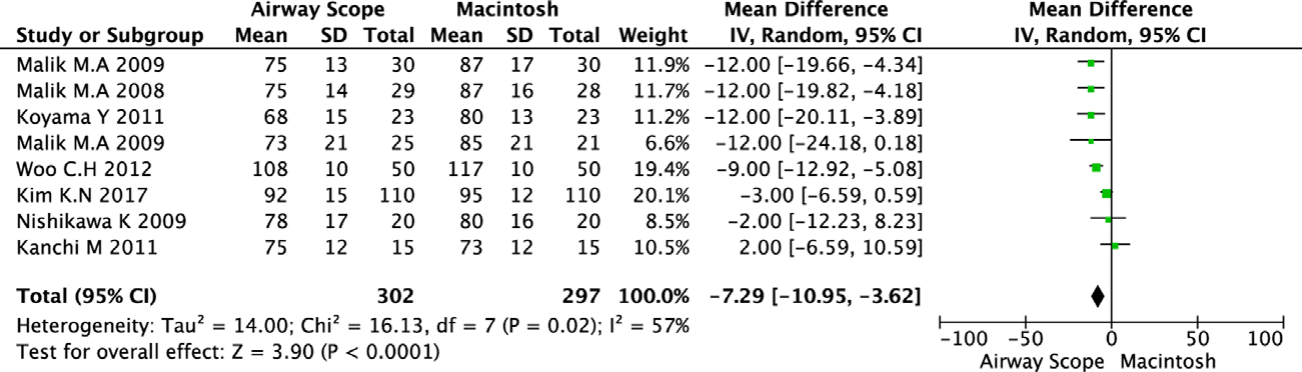
Forest plot of heart rate for tracheal intubation using the Airway Scope compared with the Macintosh laryngoscope. The center of each square represents the weighted mean difference for individual trials, and the corresponding horizontal line represents the 95% CI. The diamonds represent the pooled results. CI = confidence interval.

The Airway Scope significantly reduced HR at 60 seconds after tracheal intubation compared to the ML (WMD = −7.29; 95% CI, −10.9 to −3.62; *P* < .0001; *I*^2^ = 57%, Cochrane *Q* = 16.1) (Fig. 2). From the TSA, the 95% CI was adjusted to −11.3 to −3.3. TSA further showed the accrued information size (n = 203) to be 91.4% of the previously estimated RIS (n = 222).

**Figure 3.**
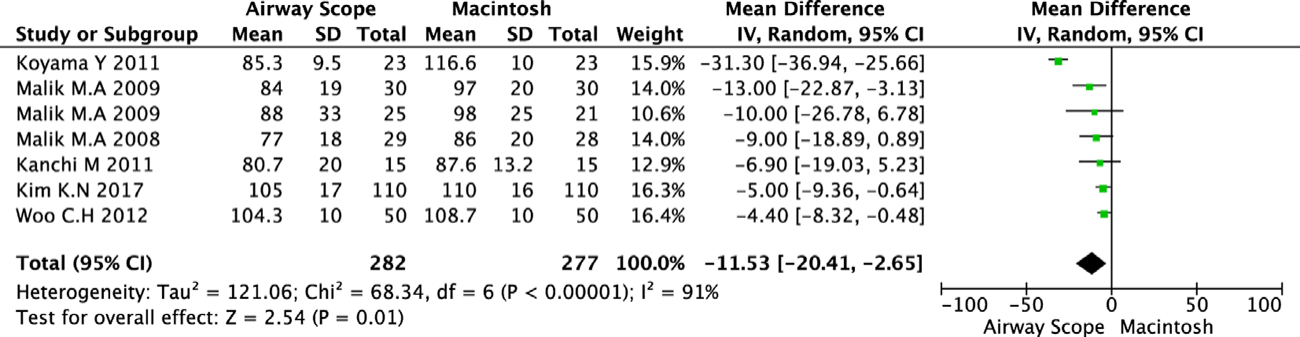
Forest plot of mean blood pressure for tracheal intubation using the Airway Scope compared with the Macintosh laryngoscope. The center of each square represents the weighted mean difference for individual trials, and the corresponding horizontal line represents the 95% CI. The diamonds represent the pooled results. CI = confidence interval.

MBP at 60 seconds after tracheal intubation was recorded in 7 studies, and the Airway Scope significantly reduced MBP at 60 seconds after tracheal intubation compared to the ML (WMD = −11.5; 95% CI, −20.4 to −2.65; *P* = .01; *I*^2^ = 91%, Cochrane Q = 68.3) (Fig. 3). However, the TSA showed that only 12.4% (n = 599) of the RIS was achieved (n = 4821).

#### 3.2.2. Secondary outcome.

Although the Airway Scope significantly reduced HR at 120 seconds and 180 seconds after tracheal intubation (120 seconds: trial [n] = 5; WMD = −7.05; 95% CI, −10.7 to −3.42; *P* = .0001; *I*^2^ = 0.0%, Cochrane Q = 3.74; 180 seconds: trial [n] = 5; WMD = −8.95; 95% CI, −15.9 to −1.94; *P* = .01; *I*^2^ = 70%, Cochrane *Q* = 13.3), it did not significantly reduce HR at 300 seconds after tracheal intubation. Conversely, the Airway Scope did not significantly reduce MBP at 120 seconds, 180 seconds, and 300 seconds after tracheal intubation (Table 2).

**Table 2:** The results of hemodynamic responses comparing heart rate or mean blood pressure in tracheal intubation using the Airway Scope and the Macintosh laryngoscope.

	Number of trials	WMD (95% CI)	*P* value	Heterogeneity test	
				I2, %	Cochrane Q
HR					
**Primary outcome**					
60 s after intubation	8	−7.29 (−10.9 to −3.62)	<.0001	57	16.1
**Secondary outcome**					
120 s after intubation	5	−7.05 (−10.7 to −3.42)	.0001	0.0	3.74
180 s after intubation	5	−8.95 (−15.9 to −1.94)	.01	70	13.3
300 s after intubation	8	−2.77 (−5.61 to 0.06)	.06	36	11.0
MBP					
**Primary outcome**					
60 s after intubation	7	−11.5 (−20.4 to −2.65)	.01	91	68.3
**Secondary outcome**					
120 s after intubation	5	−6.98 (−14.0 to 0.06)	.05	56	34.3
180 s after intubation	6	−3.22 (−7.31 to 0.86)	.12	45	9.08
300 s after intubation	7	−1.30 (−5.22 to 2.62)	.54	49	12.1

CI = confidence interval, HR = heart rate, MBP = mean blood pressure, WMD = weighted mean difference.

#### 3.2.3. Sensitivity analysis.

We performed a sensitivity analysis using a multivariate random-effects model. The results were the same for our primary and secondary outcomes. Details are shown in Table 3.

**Table 3:** The sensitive analysis of hemodynamic responses comparing heart rate or mean blood pressure in tracheal intubation using the Airway Scope and the Macintosh laryngoscope.

	MD (95% CI)	*P* value
HR		
60 s after intubation	−8.85 (−13.2 to −4.49)	<.0001
120 s after intubation	−6.55 (−11.0 to −2.10)	.004
180 s after intubation	−8.46 (−14.8 to −2.09)	.009
300 s after intubation	−2.98 (−6.93 to 0.97)	.14
MBP		
60 s after intubation	−13.3 (−21.8 to −4.69)	.002
120 s after intubation	−5.19 (−11.3 to 0.99)	.090
180 s after intubation	−3.09 (−6.83 to 0.65)	.10
300 s after intubation	−0.54 (−3.52 to 2.44)	.72

CI = confidence interval, HR = heart rate, MBP = mean blood pressure, WMD = mean difference.

### 3.3. Results of quality of evidence

**Figure 4.**
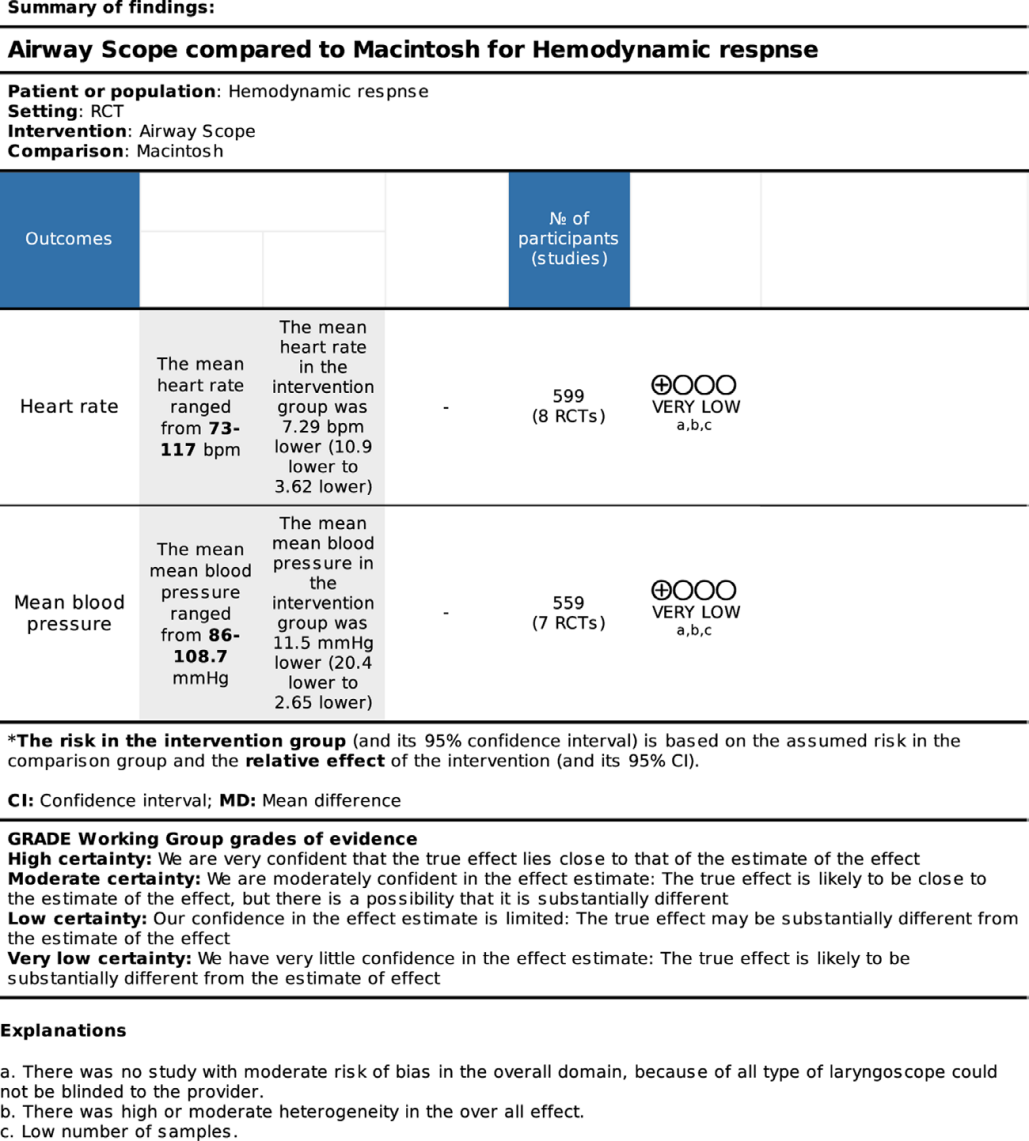
The grading of recommendations assessment, development and evaluation (GRADE) approach.

The quality of the evidence in this meta-analysis was graded as low or very low. All studies used in this meta-analysis have moderate bias, as laryngoscopists were unable to blind the type of laryngoscope used to perform tracheal intubation. Moreover, the number of samples included in this analysis was fewer than the RIS. The result of our meta-analyses at HR showed moderate heterogeneity, and meta-analyses at MBP showed high heterogeneity (Fig. 4).

### 3.4. Result of publication bias

**Figure 5.**
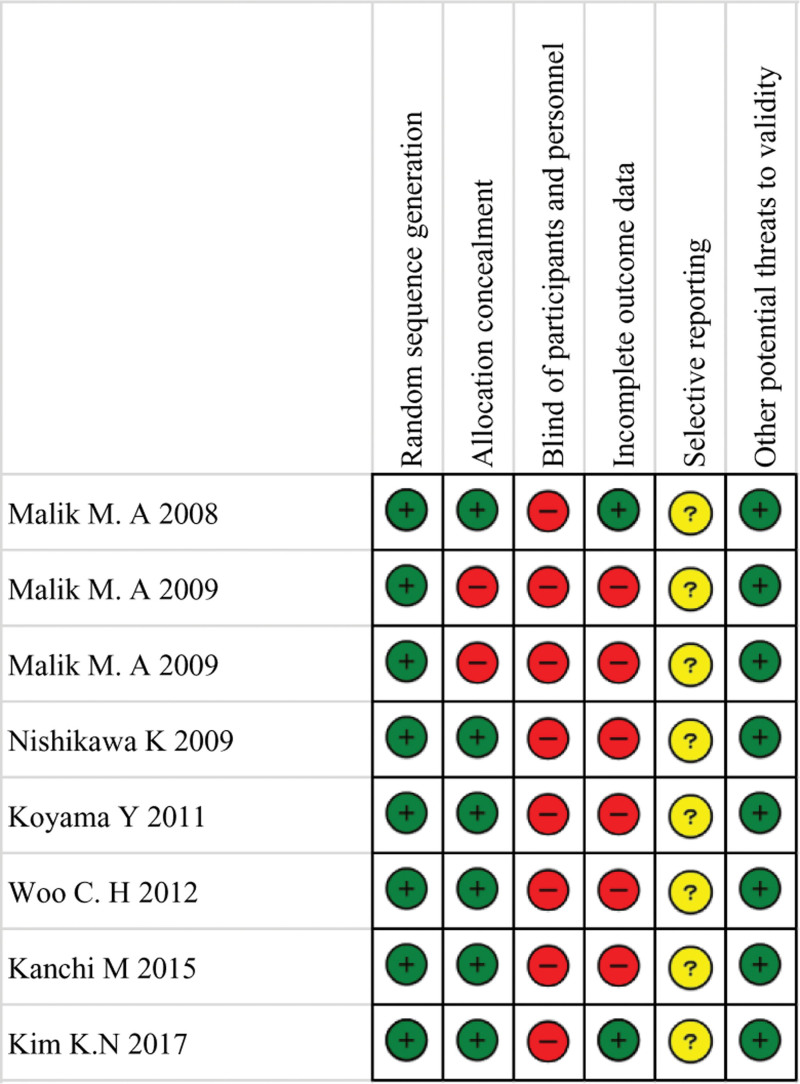
Green circles, red circles, and yellow circles indicate “low risk of bias,” “high risk of bias,” and “unclear risk of bias,” respectively.

Publication bias was not evaluated because the number of studies included in the analysis was small (<10). The risks of bias are summarized in Figure 5.

## 4. Discussion

### 4.1. Explanation of results

This study showed that compared to the ML, the Airway Scope reduced hemodynamic response at 60 seconds after tracheal intubation. These results were supported by the sensitivity analysis. TSA suggested that the need for additional studies to confirm the results for MBP.

Generally, during and after tracheal intubation, changes in hemodynamics such as hypertension and tachycardia occur. The mechanical stimulation of the upper airways induced by laryngoscopy and intubation is the primary cause of the hemodynamic response occurring with tracheal intubation.^[6,7]^ When the ML is used to intubate the trachea, the tongue and epiglottis need to be displaced by the blade with an upward lifting force, which aligns the oral-pharyngeal-tracheal axes to produce a good line of sight. The maximal force applied to the base of the tongue can be as high as 30 to 50 N when the ML is used.^[36–39]^ A much lower force is exerted by the Airway Scope, and glottic visualization can be achieved without alignment of the anatomical axes. Goto et al^[37]^ used a high-fidelity simulator to evaluate the forces applied to the tongue by the Airway Scope and the ML during intubation. They found that the Airway Scope facilitated intubation by requiring less force on the tongue than that applied with the ML (11 N vs 27 N, *P* < .001). As well, the structure of the blade in this device might help to reduce the hemodynamic response. The design of the Airway Scope differs from that of the conventional blade because it is based on the Oro pharyngeal anatomy. The wide area of the anatomically designed blade lifts the Oro pharyngeal structure, thus reducing the amount of force applied per unit area. We therefore assumed that the Airway Scope blade facilitated significantly less painful manipulation, thus leading to hemodynamic stabilization during intubation.

Procedural differences in the use of the 2 laryngoscopes during tracheal intubation may be another potential reason for the hemodynamic stabilization. A tube-guiding channel facilitates intubation in the Airway Scope. Therefore, the trachea can be easily intubated as the tracheal tube is advanced, while maintaining an approach angle of the tube tip that is aligned with the tracheal axis. As reported by Kazama et al^[40]^, stimulation during tracheal intubation can be more strongly affected when the tracheal tube is inserted into the trachea.

Conversely, the Macintosh direct laryngoscope does not have tracheal tube guidance. Its use thus requires complex hand-eye coordination within the limited oral space because the physician must manipulate the laryngoscope to visualize the glottis with 1 hand and intubate the trachea with the other hand. This factor might also increase mechanical stimulation of the Oro pharyngeal tissue.^[41]^ Further, a rigid stylet is usually used with the ML to facilitate tracheal tube manipulation. Although the rigid stylet is usually removed after the tube tip passes the glottis, the stylet temporarily increases the intensity of the mechanical contact between the tube tip and the tracheal tissue and thus the cardiovascular response after intubation.

The Airway Scope did not significantly reduce MBP at 120 and 180 seconds after tracheal intubation compared to the ML, possibly because only a small number of samples were available for the analysis. As shown by the TSA results, the sample number for MBP was insufficient. Particularly, the sample sizes for 120 seconds and 180 seconds after tracheal intubation were less than the number of samples for the primary outcome and were insufficient for the analysis.

This study reveals that the Airway Scope reduces the hemodynamic response compared to the Macintosh laryngoscope for tracheal intubation. The fact that the Airway Scope reduces the hemodynamic response to tracheal intubation is advantageous for use during awake intubation in patients with anticipated upper airway obstruction. Additionally, Airway Scope may be advantageous for tracheal intubation in patients with circulatory instability such as severe myocardial ischemia and cerebral hemorrhage. In addition, Airway Scope has the potential to provide safer tracheal intubation for patients in situations where sufficient general anesthetic is not available in the emergency department or intensive care unit.

### 4.2. Limitations of the meta-analysis

There are some limitations to this study. First, statistical processing comparing mean blood pressure responses included only a small number of studies and patients, reducing the statistical power of the studies. Further studies with larger numbers of patients are needed to evaluate the superiority of the Airway Scope over the ML in preventing hemodynamic response after tracheal intubation. Second, all eight studies included in our meta-analysis have moderate risk of bias because the anesthesia provider unable to blind the type of laryngoscope used in any of the studies. Third, clinical and methodological differences were present due to variations in the design of the original studies, for example, the studies were a population of patients with different characteristics, the skill levels of the laryngoscopists were different, and different anesthesia methods were used. Especially, the amount of analgesic used to induction of anesthesia has a great influence on the hemodynamic responses during tracheal intubation. This is an inherent limitation of meta-analyses.

## 5. Conclusion

The present study showed that compared to the ML, the Airway Scope significantly reduced both the hemodynamic response at 60 seconds after tracheal intubation and the HR at 120 seconds and 180 seconds thereafter. However, it caused no significant reduction in MBP at 120 seconds, 180 seconds, and 300 seconds after tracheal intubation. The sensitivity analysis supported these results. As TSA suggested that additional studies should be performed to confirm the results for MBP. The quality of evidence for both outcomes was very low.

## Author contributions

**Conceptualization:** Hiroshi Hoshijima.

**Data curation:** Hiroshi Hoshijima, Takumi Nagumo, Koichi Maruyama, Tsutomu Mieda, Aiji Sato.

**Formal analysis:** Hiroshi Hoshijima, Takahiro Mihara, Toshiya Shiga.

**Investigation:** Hiroshi Hoshijima, Takumi Nagumo, Koichi Maruyama.

**Methodology**: Hiroshi Hoshijima, Koichi Maruyama, Takahiro Mihara, Tsutomu Mieda.

**Project administration:** Hiroshi Hoshijima, Takahiro Mihara.

**Resources:** Hiroshi Hoshijima, Takahiro Mihara, Aiji Sato.

**Software:** Hiroshi Hoshijima.

**Supervision:** Hiroshi Hoshijima, Toshiya Shiga, Hiroshi Nagasaka.

**Validation:** Hiroshi Hoshijima.

**Writing – original draft:** Hiroshi Hoshijima, Tsutomu Mieda, Toshiya Shiga.

**Writing – review & editing:** Hiroshi Hoshijima, Aiji Sato, Toshiya Shiga, Hiroshi Nagasaka.

## Supplementary Material






