# Supplementary material for: Hemodynamic response related to the Airway Scope versus the Macintosh laryngoscope: A systematic review and meta-analysis with trial sequential analysis
Source: Medicine (Baltimore). 2023 Feb 22;102(8):e33047. doi: 10.1097/MD.0000000000033047 (PMC11309650; doi:10.1097/MD.0000000000033047)
Supplement: Supplementary file 1 [file medi-102-e33047-s001.pdf]

Supple 1.

Search terms for PubMed

(Airwayscope[All Fields] OR pentax-aws[All Fields]) AND (‘haemodynamic’[All Fields] OR ‘hemodynamics’[MeSH Terms] OR ‘hemodynamics’[All Fields] OR ‘hemodynamic’[All Fields])

Search strategy for the Cochrane Central Register of Controlled Trials

‘videolaryngoscopy or videolaryngoscope or videolaryngoscopic or videolaryngoscopes or "video laryngoscopy" or "video laryngoscope" or "video laryngoscopic" or "video laryngoscopes" or "Airway Scope" or "AirwayScope" or "Macintosh" or "Macintosh laryngoscope" or "hemodynamic response" or "hemodynamic change" in Title, Abstract, Keywords and operation or emergent or urgent in Title, Abstract, Keywords'

Search strategy for Embase

('videolaryngoscopy':ab,ti OR 'videolaryngoscope':ab,ti OR 'video laryngoscopy':ab,ti OR 'video laryngoscope':ab,ti OR AirwayScope:ab,ti OR macintosh:ab,ti OR macintosh laryngoscope:ab,ti) AND (emergen*:ab,ti,kw OR operation*:ab,kw,ti) AND ('clinical article'/de OR 'clinical trial'/de

OR 'comparative study'/de OR 'controlled clinical trial'/de OR 'controlled study'/de OR 'crossover procedure'/de OR 'human'/de OR 'human experiment'/de OR 'intermethod comparison'/de OR 'major clinical study'/de OR 'meta analysis'/de OR 'multicenter study'/de OR 'observational study'/de OR 'prospective study'/de OR 'randomized controlled trial'/de OR 'randomized controlled trial (topic)'/de OR 'retrospective study'/de OR 'total quality management'/de)
