# Supplementary material for: Hemodynamic response related to the Airway Scope versus the Macintosh laryngoscope: A systematic review and meta-analysis with trial sequential analysis
Source: Medicine (Baltimore). 2023 Feb 22;102(8):e33047. doi: 10.1097/MD.0000000000033047 (PMC11309650; doi:10.1097/MD.0000000000033047)
Supplement: Supplementary file 2 [file medi-102-e33047-s002.pdf]

Supple 2. Risks of bias assessment

The risks of bias was estimated in the following methodological domains: sequence generation; allocation concealment; blinding of participants; incomplete outcome data; selective outcome reporting; and other potential threats to validity.
