# Supplementary material for: Hemodynamic response related to the Airway Scope versus the Macintosh laryngoscope: A systematic review and meta-analysis with trial sequential analysis
Source: Medicine (Baltimore). 2023 Feb 22;102(8):e33047. doi: 10.1097/MD.0000000000033047 (PMC11309650; doi:10.1097/MD.0000000000033047)
Supplement: Supplementary file 3 [file medi-102-e33047-s003.pdf]

Supple 3. Grading of Recommendations Assessment

The Grading of Recommendations Assessment, Development and Evaluation (GRADE) approach was applied with GRADEpro software (version 3.6 for Windows; available from http://ims.cochrane.org/revman/gradepro) to assess the quality of evidence of the main outcomes. Furthermore, the quality of evidence was based on the presence or absence of the following variables: limitations of the study design, inconsistency, indirectness, imprecision of the results and publication bias. The quality of evidence for the main outcomes was graded as very low, low, moderate or high.
